# Trends in the Prevalence of Depression in Hospitalized Patients with Type 2 Diabetes in Spain: Analysis of Hospital Discharge Data from 2001 to 2011

**DOI:** 10.1371/journal.pone.0117346

**Published:** 2015-02-23

**Authors:** Ana Lopez-de-Andrés, Mª Isabel Jiménez-Trujillo, Valentín Hernández-Barrera, José Mª de Miguel-Yanes, Manuel Méndez-Bailón, Napoleón Perez-Farinos, Carmen de Burgos Lunar, Juan Cárdenas-Valladolid, Miguel Ángel Salinero-Fort, Rodrigo Jiménez-García, Pilar Carrasco-Garrido

**Affiliations:** 1 Preventive Medicine and Public Health Teaching and Research Unit, Health Sciences Faculty, Rey Juan Carlos University, Alcorcon, Comunidad de Madrid, Spain; 2 Medicine Department, Hospital Universitario del Sureste, Arganda, Comunidad de Madrid, Spain; 3 Medicine Department, Hospital Clínico San Carlos, Madrid, Comunidad de Madrid, Spain; 4 Health Security Agency, Ministry of Health, Madrid, Comunidad de Madrid, Spain; 5 Preventive Medicine Department, Hospital La Paz, Madrid, Comunidad de Madrid, Spain; 6 Dirección Técnica de Docencia e Investigación, Gerencia Atención Primaria, Madrid, Comunidad de Madrid, Spain; Leibniz Institute for Neurobiology, GERMANY

## Abstract

**Background:**

This study aims to describe trends in the prevalence of depression among hospitalized patients with type 2 diabetes in Spain, 2001–2011.

**Methods:**

We selected patients with a discharge diagnosis of type 2 diabetes using national hospital discharge data. Discharges were grouped by depression status. Prevalence of depression globally and according to primary diagnoses based on the Charlson comorbidity index (CCI) were analyzed. We calculated length of stay (LOHS) and in-hospital mortality (IHM). Multivariate analysis was adjusted by age, year and comorbidity.

**Results:**

From 2001 to 2011, 4,723,338 discharges with type 2 diabetes were identified (4.93% with depression). Prevalence of depression in diabetic patients increased from 3.54% in 2001 to 5.80% in 2011 (p<0.05). The prevalence of depression was significantly higher in women than in men in each year studied and increased from 5.22% in 2001 to 9.24% in 2011 (p<0.01). The highest prevalence was observed in the youngest age group (35–59 years). The median LOHS decreased significantly over this period. Men with diabetes and depression had higher IHM than women in all the years studied (p<0.05). Older age and greater comorbidity were significantly associated with a higher risk of dying, among diabetic patients with concomitant depression.

**Conclusions:**

Prevalence of depression increased significantly among hospitalized diabetic patients from 2001 to 2011 even if the health profile and LOHS have improved over this period. Programs targeted at preventing depression among persons with diabetes should be reinforced in Spain.

## Introduction

The prevalence of depression is higher in patients with type 2 diabetes (T2DM), compared with those without diabetes [1–3]. In previous reviews, the prevalence of depression was found to be nearly twice higher among patients with T2DM than those without diabetes [1–3]. Epidemiological studies conducted in Spain have confirmed this finding [4, 5].

The studies linking these two comorbid conditions support the hypothesis of a bi-directional relationship between diabetes and depression, indicating that depression itself is a risk factor for the development of diabetes [6–10]. Mezuk et al conducted a meta-analysis finding that depression was associated with a 60% increased risk of T2DM (Risk Ratio 1.60, 95% CI; 1.37–1.88) whereas T2DM was associated with an only modest (RR 1.15, 95% CI 1.02–1.30) risk of depression [6].

Different authors have investigated various pathways explaining the underlying mechanisms connecting these diseases [7–10]. The increased risk of T2DM in individuals with depression could result from increased counter-regulatory hormone release and action, alterations in glucose transport function, and increased immunoinflammatory activation [7]. These physiologic alterations are thought to contribute to insulin resistance and beta islet cell dysfunction, which ultimately lead to the development of T2DM. Furthermore, depression is associated with poor health behavior, maladaptive coping style, social isolation, and chronic life stress that help the development of diabetes [8]. Depressive symptoms are associated with higher body mass index (BMI) and with unhealthy behaviors such as consuming high calorie diets, which in turn are risk factors for the development of T2DM [9].

On the other hand, when diabetes precedes depression and leads to this disease it could be a consequence of either through a direct effect of hyperglycaemia, possibly leading to altered glucose transport, or as a result of the psychological stress resulting from the knowledge of the diagnosis or from the rigours of the treatment, both lifestyle and pharmacological [10].

Since depression is associated with reduced adherence to self-care behaviors (i.e., diet, exercise, and smoking cessation) and to diabetes medication, patients may be at risk of worse health status and increased comorbidity and would therefore consume more healthcare resources [5, 11–18]. Gonzalez et al. found that the presence of depressive symptoms are good predictors of poor adherence to medications and diet and exercise regimens among T2DM patients [19]. Vamos *et al*. concluded that hospital stay was significantly longer in diabetic patients with comorbid depression than in those who did not have depression (13.5 days vs. 6.0 days; p<0.001) [14]. In Spain, a study conducted among patients with T2DM patients aged 35 years and over in the Basque Country estimated that patients suffering concomitant depression had an average cost per patient/year 516€ higher than patients with just T2DM (p<0.001) adjusted by the other covariates [5].

Concurrent diabetes and depression is also associated with significantly greater all-cause mortality than either diabetes or depression alone [15–18]. Egede *et al*. showed a hazard ratio (HR) for all-cause mortality of 1.88 (95%CI, 1.55–2.27) in individuals with diabetes only, compared to 2.50 (95%CI, 2.04–3.08) in individuals with coexisting diabetes and depression [17].

To our knowledge, no authors have investigated national trends in the prevalence of depression in hospitalized patients with T2DM. In this study, we used national hospital discharge data to describe trends in the prevalence of depression among hospitalized patients with T2DM between 2001 and 2011 in Spain. We analyzed the prevalence of depression according to specific admission diagnoses and in-hospital outcomes such as in-hospital mortality (IHM) and length of hospital stay (LOHS).

## Materials and Methods

We performed a retrospective, observational study using the Spanish National Hospital Database (CMBD, *Conjunto Minimo Básico de Datos*), which is managed by the Spanish Ministry of Health and Social Policy and Equality and compiles all public and private hospital data, thus covering more than 95% of hospital discharges [11]. The Spanish National Hospital Database includes demographic variables (sex, date of birth), date of admission, date of discharge, up to 14 discharge diagnoses, and up to 20 procedures performed during admission. The Spanish Ministry of Health and Social Policy and Equality sets standards for registration and performs regular audits [20]. We analyzed data collected between January 1, 2001 and December 31, 2011.

Disease criteria were defined according to the International Classification of Diseases-Ninth Revision, Clinical Modification (ICD-9-CM), which is used in the Spanish CMBD. We selected all admission diagnoses of patients aged ≥ 35 years with T2DM (ICD-9-MC codes 250.x0 or 250.x2) identified based on any diagnosis field.

Discharges were grouped by depression status. Depression was classified as the presence of ICD-9-CM codes 300.4, 301.12, 309.1, and 311 in any diagnosis field.

We analyzed the prevalence of depression and the outcome of hospitalization globally and according to a series of primary diagnoses based on the disease categories included in the Charlson comorbidity index, as follows: myocardial infarction (410.x and 412.x), congestive heart failure (398.91, 402.01, 402.11, 402.91, 404.01, 404.03, 404.11, 404.13, 404.91, 404.93, 425.4–425.9, and 428.x), peripheral vascular disease (093.0, 437.3, 440.x, 441.x, 443.1–443.9, 47.1, 557.1, 557.9, and V43.4), cerebrovascular disease (362.34 and 430.x-438.x), chronic pulmonary disease (416.8, 416.9, 490.x-505.x, 506.4, 508.1, and 508.8), renal disease (403.01, 403.11, 403.91, 404.02, 404.03, 404.12, 404.13, 404.92, 404.93, 582.x, 583.0–583.7, 585.x, 586.x, 588.0, V42.0, V45.1, and V56.x), any malignancy (including lymphoma and leukemia), except malignant neoplasm of the skin (140.x-172.x, 174.x-195.8, 200.x-208.x, and 238.6), moderate or severe liver disease (456.0–456.2 and 572.2–572.8), and metastatic solid tumor (196.x-199.x)[12].

The outcomes of interest included LOHS and the percentage of patients who died during admission, which we defined as IHM.

### Statistical analysis

We calculated the prevalence of depression in patients with T2DM by age group and according to the primary diagnosis by dividing the number of cases of depression each year by the corresponding number of patients with T2DM in each subgroup and year. All results are presented separately for women and men.

Variables are shown as percentages and means with standard deviation or medians with the interquartile range. Bivariate analysis of variables according to the year was conducted using binary logistic regression (proportions) and ANOVA or the Kruskal-Wallis test (medians), as appropriate. We used a non parametric test for LOHS because this variable is not normally distributed according to the Kolmogorov-Smirnov Test.

To assess the time trend in prevalence we used logistic regression analysis adjusted for age.

In order to test the time trend for IHM adjusted for all other variables, logistic regression analysis was performed with mortality as a binary outcome and year of discharge, sex, age, and Charlson comorbidity index as independent variables. The Charlson comorbidity index applies to 17 disease categories whose scores are totaled to obtain an overall score for each patient. A detailed description of the approach is available elsewhere [21].

The statistical analysis was performed using Stata version 10.1 (Stata, College Station, Texas, USA). Statistical significance was set at p<0.05 (2-tailed).

### Ethics

Data confidentiality was maintained at all times according to Spanish legislation. Patient identifiers were deleted before the database was provided to the authors in order to maintain anonymity. It is not possible to identify patients either in this article or in the database. Given the anonymous and mandatory nature of the dataset, it was not necessary to obtain informed consent. The study protocol was approved by the Ethics Committee of Rey Juan Carlos University.

## Results

During the study period, 4,723,338 patients aged ≥35 years had a diagnosis of T2DM; the codes for depression were included in the discharge diagnosis codes in 233,026 (4.93%) patients. The characteristics of hospital discharges for patients with T2DM and depression in Spain from 2001–2011 are shown in Table 1. The proportion of men increased significantly form 27.72% to 28.93% and the mean age raised from 69.81 to 72.40 years (p<0.05). Depression was recorded as the main diagnosis in a very low proportion of discharged diabetic patients (2.39% in 2001 and 1.30% in 2011). The most common ICD-9 CM code used was 311 (Depressive disorder, not elsewhere classified) appearing in over two thirds of the subjects.

**Table 1:** Characteristics of the type 2 diabetic patients suffering depression discharged from Spanish hospitals according to year.

		2001	2002	2003	2004	2005	2006	2007	2008	2009	2010	2011
N *		10548	11895	16030	17855	19705	21007	23261(100)	25358(100)	27405	28657	31305
Male *	N (%)	2924(27.72)	3434(28.87)	4589(28.63)	5075(28.42)	5536(28.09)	5826(27.73)	6773(29.12)	7180(28.31)	7637(27.87)	8163(28.49)	9056(28.93)
Age *	Mean (SD)	69.81(10.74)	70.12(10.69)	70.61(10.73)	70.84(10.86)	71.42(10.83)	71.53(11.06)	71.73(11.01)	72.06(11.24)	72.01(11.35)	72.21(11.34)	72.40(11.44)
Depression as main diagnosis *	N (%)	252(2.39)	276(2.32)	292(1.82)	290(1.62)	300(1.52)	340(1.62)	319(1.37)	372(1.47)	354(1.29)	395(1.38)	408(1.30)
Depression ICD9 CM Code N (%) *	300.4	2544(24.12)	2930(24.63)	4129(25.76)	4824(27.02)	5282(26.81)	6089(28.99)	6628(28.49)	7170(28.28)	8200(29.92)	8955(31.25)	9954(31.8)
	301.12	6(0.06)	3(0.03)	11(0.07)	6(0.03)	7(0.04)	6(0.03)	16(0.07)	26(0.1)	20(0.07)	9(0.03)	10(0.03)
	309.1	144(1.37)	152(1.28)	254(1.58)	297(1.66)	304(1.54)	252(1.2)	280(1.2)	383(1.51)	377(1.38)	455(1.59)	490(1.57)
	311	7868(74.59)	8823(74.17)	11653(72.69)	12754(71.43)	14146(71.79)	14706(70.01)	16387(70.45)	17837(70.34)	18867(68.85)	19305(67.37)	20923(66.84)
Number of comorbidities N (%)*	None	4305(40.81)	4717(39.66)	5992(37.38)	6631(37.14)	7227(36.68)	7734(36.82)	8484(36.47)	9134(36.02)	9744(35.56)	10096(35.23)	11009(35.17)
	1 or 2	4818(45.68)	5572(46.84)	7712(48.11)	8504(47.63)	9439(47.9)	10041(47.8)	11210(48.19)	12063(47.57)	13122(47.88)	13443(46.91)	14492(46.29)
	3 or more	1425(13.51)	1606(13.5)	2326(14.51)	2720(15.23)	3039(15.42)	3232(15.39)	3567(15.33)	4161(16.41)	4539(16.56)	5118(17.86)	5804(18.54)

*p<0.05 (Time trend analysis). ICD9 codes: 300.4 “Dysthymic disorder”; 301.12 “Chronic depressive personality disorder”; 309.1 “Prolonged depressive reaction”; 311 “Depressive disorder, not elsewhere classified”

**Fig 1:**
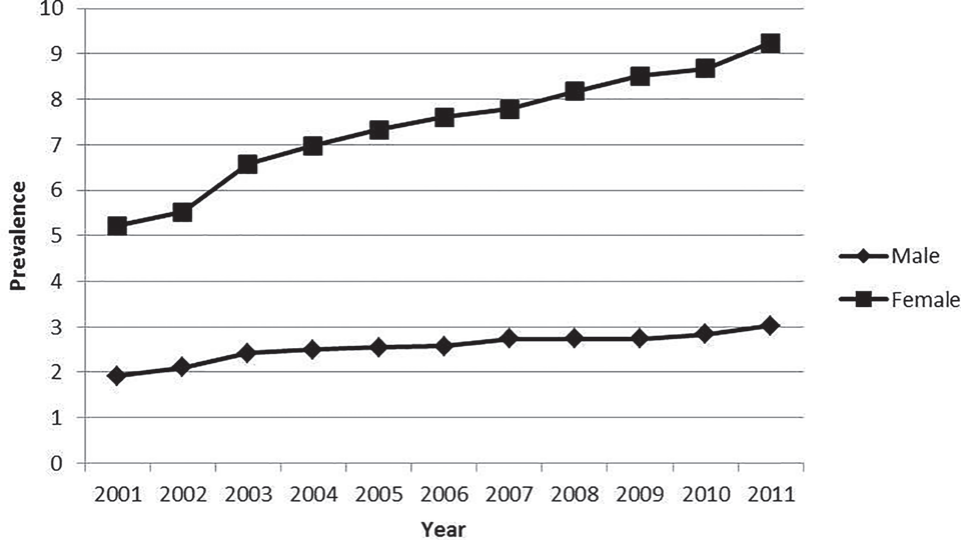
Prevalence of depression among men and women suffering type 2 diabetes by year. Prevalence: Prevalence of depression among patients with type 2 diabetes.

Prevalence of depression in patients with T2DM increased significantly during the study period, from 3.54% in 2001 to 5.80% in 2011 (p<0.05). Fig. 1 shows the prevalence of depression among men and woman suffering T2DM subjects by year. The prevalence of depression was significantly higher in women in every single year studied and increased from 5.22% in 2001 to 9.24% in 2011 (p<0.01). The prevalence of depression in diabetic men increased from 1.92% to 3.03% (p<0.01).

The distribution and outcomes of hospital discharges for patients with T2DM and depression in Spain from 2001–2011according to sex are shown in Table 2. For both diabetic men and women, the prevalence of depression increased significantly in all age groups during the study period. For both sexes, the highest prevalence was observed in the youngest age group (35–59 years), although this fell with age.

**Table 2:** Prevalence of depression among hospitalized patients with type 2 diabetes in Spain 2001–2011, according to age and sex.

		2001	2002	2003	2004	2005	2006	2007	2008	2009	2010	2011
Male	N	152287	162994	189766	203010	217539	227123	246845	262716	279598	288739	299346
	Prevalence* (95% CI)	1.92 (1.86–1.98)	2.11 (2.05–2.17)	2.42 (2.36–2.48)	2.5 (2.44–2.56)	2.54 (2.49–2.6)	2.57 (2.51–2.62)	2.74 (2.69–2.8)	2.73 (2.68–2.79)	2.73 (2.68–2.78)	2.83 (2.78–2.88)	3.03 (2.97–3.08)
	35–59year N (%)*	28282 (1.93)	30260 (2.22)	34410 (2.62)	36686 (2.72)	38419 (2.74)	39715 (2.86)	42330 (2.93)	44139 (3.32)	46687 (3.21)	47098 (3.42)	48640 (3.8)
	60–69 year N (%)*	42736 (1.89)	43606 (1.89)	49156 (2.34)	51218 (2.51)	53466 (2.55)	55307 (2.57)	57931 (2.75)	62617 (2.68)	68254 (2.68)	70676 (2.88)	73749 (2.99)
	70–79 year N (%)*	57759 (1.99)	62335 (2.27)	73374 (2.36)	78538 (2.44)	83758 (2.45)	86435 (2.45)	93448 (2.71)	96989 (2.58)	100613 (2.58)	101870 (2.56)	102685 (2.78)
	≥80 years N (%)*	23510 (1.81)	26793 (1.95)	32826 (2.46)	36568 (2.41)	41896 (2.56)	45666 (2.53)	53136 (2.64)	58971 (2.61)	64044 (2.68)	69095 (2.77)	74272 (2.89)
	LOHS* Median (IQR)	8 (5–15)	8(5–15)	8(5–14)	8(5–14)	8(4–14)	8(4–14)	8(4–13)	7(4–13)	7(4–12)	7(4–12)	7(3–12)
	IHM, % (95% CI)	5.95 (5.23–6.67)	6.03 (5.36–6.69)	6.47 (5.88–7.07)	6.23 (5.67–6.78)	6.95 (6.39–7.52)	6.63 (6.09–7.16)	6.27 (5.79–6.76)	6.25 (5.78–6.72)	6.26 (5.8–6.71)	5.73 (5.31–6.16)	5.93 (5.52–6.34)
Female	N	146079	153373	174170	183202	193336	199484	211803	222423	232200	236490	240815
	Prevalence* (95% CI)	5.22 (5.12–5.31)	5.52 (5.42–5.61)	6.57 (6.47–6.67)	6.98 (6.88–7.07)	7.33 (7.23–7.43)	7.61 (7.51–7.71)	7.78 (7.69–7.88)	8.17 (8.08–8.27)	8.51 (8.42–8.61)	8.67 (8.57–8.76)	9.24 (9.14–9.34)
	35–59 year N (%)*	16421 (7.67)	17026 (7.84)	18688 (8.85)	19285 (9.55)	19288 (9.57)	19985 (10.24)	20736 (10.55)	21373 (10.4)	22424 (11.7)	22288 (11.43)	23012 (12.28)
	60–69 year N (%)*	32350 (6.25)	32167 (6.65)	34562 (7.87)	35025 (8.42)	35241 (8.82)	34902 (8.99)	34983 (9.68)	36391 (10.28)	38099 (10.51)	38276 (10.91)	38276 (11.36)
	70–79 year N (%)*	55908 (5.1)	58864 (5.67)	66776 (6.92)	70574 (7.09)	73802 (7.58)	75654 (7.7)	79715 (7.94)	81033 (8.32)	82120 (8.79)	81530 (9.1)	79565 (9.71)
	≥80 years N (%)*	41400 (3.60)	45316 (3.64)	54144 (4.52)	58318 (5.12)	65005 (5.57)	68943 (6.05)	76369 (6.00)	83626 (6.54)	89557 (6.61)	94396 (6.72)	99962 (7.35)
	LOHS* Median (IQR)	9(5–14)	8(5–14)	8(5–13)	8(4–13)	8(4–13)	8(4–12)	7(4–13)	7(4–12)	7(4–12)	7(4–11)	7(4–11)
	IHM, %* (95% CI)	4.3 (3.92–4.68)	4.36 (4.0–4.73)	4.89 (4.56–5.22)	4.54 (4.24–4.84)	4.95 (4.66–5.25)	5.01 (4.72–5.3)	5.12 (4.84–5.4)	4.94 (4.68–5.2)	4.74 (4.49–4.99)	5.05 (4.8–5.3)	4.89 (4.66–5.13)

N: number of patients with type 2 diabetes. Prevalence: Prevalence of depression among patients with type 2 diabetes. LOHS: median of length of hospital stay due to depression among patients with type 2 diabetes. IQR: Inter quartile range. IHM: in-hospital mortality due to depression among patients with type 2 diabetes. CI: Confidence interval*p<0.05 (Time trend analysis)

Length of hospital stay and in-hospital mortality among those suffering depression.

The median LOHS was similar in both sexes (7–8 days) and decreased significantly during the study period. Men with T2DM and depression had higher IHM than women in all the years studied (p<0.05). IHM increased significantly in women with T2DMs and depression (4.30% in 2001 vs. 4.89% in 2011; p<0.05), although no significant change was observed in diabetic men (5.95% in 2001 vs. 5.73% in 2011; p<0.05).

Table 3 shows the annual prevalence of depression among men with T2DM based on the principal diagnosis at discharge. The highest prevalence of depression was found among T2DMpatients with concomitant chronic pulmonary disease and the lowest among those with peripheral vascular disease.

**Table 3:** Prevalence of depression among men hospitalized with type 2 diabetes in Spain 2001–2011, according to selected primary diagnosis, and in-hospital mortality among those suffering depression.

		2001	2002	2003	2004	2005	2006	2007	2008	2009	2010	2011
MI	N Prevalence*	6803 (1.25)	7854 (1.4)	9122 (1.72)	9539 (1.92)	9882 (1.81)	9541 (1.86)	9687 (1.61)	9902 (2.05)	10124 (2.03)	10195 (2.14)	10213 (2.32)
	IHM	10.59	8.18	14.65	10.93	10.61	8.47	8.97	8.37	9.71	9.17	6.33
CHF	N Prevalence*	8764 (1.99)	9302 (1.94)	11193 (2.02)	12686 (2.39)	13718 (2.19)	14436 (2.47)	16589 (2.46)	17372 (2.33)	18551 (2.33)	20024 (2.12)	20770 (2.5)
	IHM	9.2	6.67	9.29	6.93	7.67	8.4	7.84	6.17	8.08	6.35	7.31
PVD	N Prevalence*	3746 (1.07)	4091 (1.1)	4565 (1.34)	5168 (1.74)	5291 (1.63)	5684 (1.48)	5773 (1.56)	6522 (1.38)	6999 (1.53)	7190 (1.72)	7245 (1.74)
	IHM	5.00	6.67	6.56	3.33	3.49	8.33	3.33	2.22	4.67	4.03	3.97
CD	N Prevalence*	10512 (1.92)	10908 (2.67)	12073 (2.61)	12594 (2.69)	13128 (2.95)	13445 (2.81)	14368 (3.09)	15075 (2.99)	15248 (3.26)	15584 (3.17)	15704 (2.98)
	IHM	8.42	8.93	5.4	6.49	5.43	9.79	7.43	8.89	6.64	7.09	5.98
CPD	N Prevalence*	9729 (2.51)	11524 (2.96)	14150 (3.2)	14048 (3.2)	16315 (3.14)	12484 (3.49)	14181 (3.69)	14158 (3.18)	14798 (3.57)	14381 (3.33)	14874 (3.68)
	IHM	3.28	5.28	5.08	4.44	6.43	5.5	4.4	4.22	3.41	2.92	5.29
RD	N Prevalence	1068 (2.62)	1132 (2.39)	1337 (1.72)	1473 (2.51)	1599 (1.88)	1682 (2.5)	1933 (2.12)	1500 (2.2)	1331 (2.33)	1452 (1.72)	1414 (2.19)
	IHM	7.14	7.41	8.7	10.81	23.33	0	7.32	6.06	6.45	4	0
Cancer	N Prevalence*	12288 (1.72)	12832 (1.58)	15055 (2)	16809 (2.24)	17747 (2.34)	18715 (2.11)	20302 (2.27)	22215 (2.4)	24434 (2.38)	26024 (2.48)	27451 (2.83)
	IHM	9.48	12.81	14.95	13.53	16.63	14.72	12.15	13.51	14.8	13.31	12.85
LD	N Prevalence*	977 (0.72)	1031 (0.39)	1216 (0.99)	1265 (1.03)	1337 (1.42)	1489 (1.75)	1565 (1.66)	1743 (2.29)	1922 (1.87)	1754 (2)	1844 (2.28)
	IHM	14.29	0	16.67	7.69	5.26	11.54	11.54	15	19.44	11.43	4.76
MSD	N Prevalence*	1321 (1.59)	1476 (1.96)	1697 (2.18)	1925 (2.39)	2098 (2.72)	2108 (2.23)	2229 (2.92)	2512 (2.35)	2721 (2.35)	2920 (2.43)	2987 (2.68)
	IHM	9.52	34.48	32.43	19.57	28.07	38.3	20	25.42	21.88	21.13	18.75

N: number of patients with type 2 diabetes and with comorbidity (MI: myocardial infarction. CHF: congestive heart failure. PVD: peripheral vascular disease. CD: cerebrovascular disease. CPD: chronic pulmonary disease. RD: renal disease. Cancer: any malignancy, including lymphoma and leukemia, except malignant neoplasm of skin. LD: moderate o severe liver disease. MSD: metastatic solid tumor)

Prevalence: Prevalence of depression among patients with type 2 diabetes and with comorbidity. IHM: in-hospital mortality due to depression among patients with type 2 diabetes and with comorbidity

*p<0.05 (Time trend analysis adjusted by age)

After adjustment for age, we found that the prevalence of depression among diabetic men increased significantly when the primary admission diagnosis was myocardial infarction (1.25% in 2001 vs. 2.32% in 2011;p<0.05), congestive heart failure (1.99% in 2001 vs. 2.5% in 2011; p<0.05), peripheral vascular disease (1.07% in 2001 vs. 1.74% in 2011; p<0.05), cerebrovascular disease (1.92% in 2001 vs. 2.98% in 2011; p<0.05), chronic pulmonary disease (2.51% in 2001 vs. 3.68% in 2011; p<0.05), moderate or severe liver disease (0.72% in 2001 vs. 2.28% in 2011; p<0.05), any malignancy (including lymphoma and leukemia) (1.72% in 2001 vs. 2.83% in 2011; p<0.05), and metastatic solid tumor (1.59% in 2001 vs. 2.68% in 2011; p<0.05).

As expected, the highest IHM was found among men with a metastatic solid tumor and the lowest in peripheral vascular disease. However, we found no significant change over time for IHM among diabetic men with depression, according to the primary diagnosis analyzed.

The prevalence of depression was higher in women than in men; the highest prevalence was in concomitant chronic pulmonary disease (as in men). After adjustment for age (Table 4), the prevalence of depression increased significantly for the same medical diagnosis as diabetic men. The highest IHM was found among women with metastatic solid tumor. During the study period, IHM decreased significantly in women with depression when the primary diagnosis of hospitalization was myocardial infarction (13.64% in 2001 vs. 9.95% in 2011;p<0.05) and chronic pulmonary disease (2.41% in 2001 vs. 2.33% in 2011;p<0.05); however, when the principal diagnosis of hospitalization was any malignancy, including lymphoma and leukemia, the IHM increased significantly (9.84% in 2001 vs. 13.55% in 2011).

**Table 4:** Prevalence of depression among women hospitalized with type 2 diabetes in Spain 2001–2011, according to selected primary diagnosis, and in-hospital mortality among those suffering depression.

		2001	2002	2003	2004	2005	2006	2007	2008	2009	2010	2011
MI	N Prevalence*	4966 (3.99)	5587 (3.67)	6453 (4.7)	6562 (4.71)	6454 (4.74)	6020 (5.42)	6161 (5.06)	6143 (5.19)	6066 (5.75)	5742 (6.25)	5659 (6.93)
	IHM*	13.64	13.66	14.19	12.94	12.42	9.2	9.29	9.09	9.74	6.96	9.95
CHF	N Prevalence*	12955 (4.83)	13433 (5.11)	15967 (6.05)	17562 (6.29)	18777 (6.77)	19340 (7.06)	21281 (6.52)	22294 (7.45)	23109 (7.17)	24039 (7.54)	24654 (8.01)
	IHM	4.15	5.98	6.21	4.71	6.84	6.74	6.34	5.29	5.37	6.46	5.21
PVD	N Prevalence*	1662 (3.01)	1709 (3.16)	1948 (3.44)	2102 (4.33)	2134 (4.55)	2285 (4.77)	2317 (5.14)	2560 (5.66)	2672 (5.09)	2678 (5.79)	2887 (5.89)
	IHM	2	7.41	8.96	10.99	8.25	11.01	8.4	5.52	10.29	10.32	9.41
CD	N Prevalence*	10274 (5.34)	10300 (5.8)	11235 (6.94)	11564 (6.79)	11769 (7.63)	11980 (8.25)	12018 (8.08)	12810 (8.02)	12948 (8.56)	12938 (8.41)	12726 (8.71)
	IHM	6.38	6.03	8.85	6.11	9.47	9.82	8.55	8.66	7.49	8.09	8.57
CPD	N Prevalence*	4364 (6.67)	5108 (7.52)	6265 (9.47)	6169 (10.21)	6909 (10.62)	4943 (11.49)	5525 (11.58)	5573 (11.65)	5528 (12.92)	5372 (11.34)	5472 (12.55)
	IHM*	2.41	2.08	3.88	3.02	3.68	1.41	2.34	2.31	2.94	1.81	2.33
RD	N Prevalence	1187 (3.62)	1135 (4.05)	1242 (6.68)	1445 (6.3)	1426 (5.68)	1569 (6.12)	1740 (5.63)	1006 (6.06)	882 (6.92)	898 (5.57)	806 (5.09)
	IHM	2.33	6.52	3.61	6.59	7.41	8.33	4.08	1.64	8.2	6	0
Cancer	N Prevalence*	7620 (4.8)	7974 (5.51)	9068 (5.49)	9426 (6.02)	9991 (6.33)	10402 (6.57)	10882 (7.15)	11588 (7.89)	12331 (7.7)	13061 (8.03)	13350 (9.01)
	IHM*	9.84	12.98	8.84	12.52	10.28	9.66	12.34	11.6	11.28	13.63	13.55
LD	N Prevalence*	513 (2.34)	534 (3.75)	535 (2.24)	649 (5.86)	618 (4.69)	715 (5.03)	741 (5.4)	786 (5.6)	806 (6.33)	803 (4.61)	733 (6.55)
	IHM	33.33	10	25	10.53	6.9	11.11	0	9.09	9.8	10.81	10.42
MSD	N Prevalence*	1228 (3.75)	1301 (5.84)	1403 (6.41)	1589 (6.42)	1608 (6.47)	1684 (5.88)	1703 (7.69)	1817 (7.87)	1890 (7.62)	1969 (7.36)	2016 (7.79)
	IHM	21.74	23.68	23.33	20.59	25.96	20.2	22.14	21.68	20.14	25.52	22.93

N: number of patients with type 2 diabetes and with comorbidity (MI: myocardial infarction. CHF: congestive heart failure. PVD: peripheral vascular disease. CD: cerebrovascular disease. CPD: chronic pulmonary disease. RD: renal disease. Cancer: any malignancy, including lymphoma and leukemia, except malignant neoplasm of skin. LD: moderate o severe liver disease. MSD: metastatic solid tumor)

Prevalence: Prevalence of depression among patients with type 2 diabetes and with comorbidity. IHM: in-hospital mortality due to depression among patients with type 2 diabetes and with comorbidity.

*p<0.05 (Time trend analysis adjusted by age).

The results of the multivariate analysis to identify factors associated with the presence of depression in patients with T2DM and IHM are shown in Table 5.

As seen in the table and detected in the bivariate analysis, older age and having a higher score in the Charlson comorbidity index were protective factors for prevalence of depression in both men and women. After controlling for possible confounders, we observed that the prevalence of depression in patients with T2DM increased significantly over time in men and women. The adjusted increase in prevalence is greater in women than in men (OR for year 2011 vs 2001, 1.99 [95%CI, 1.93–2.04]; and OR 1.61 [95%CI, 1.55–1.68], respectively).

**Table 5:** Multivariate analysis of the factors associated with the prevalence of depression among men and women hospitalized with type 2 diabetes and in-hospital mortality among patients with type 2 diabetes and concomitant depression, Spain 2001–2011.

		Prevalence of depression		IHM among patients with type 2 diabetes and depression	
		Male OR(95%CI)	Female OR(95%CI)	Male OR(95%CI)	Female OR(95%CI)
Age	35–59 years	1	1	1	1
	60–69 years	0.87 (0.85–0.89)	0.91 (0.89–0.92)	1.57 (1.38–1.79)	1.55 (1.38–1.76)
	70–79 years	0.85 (0.83–0.87)	0.76 (0.75–0.77)	2.27 (2.01–2.55)	2.67 (2.39–2.97)
	>80 years	0.86 (0.84–0.88)	0.55 (0.54–0.56)	3.90 (3.46–4.39)	5.05 (4.53–5.63)
CCI	Units	0.97 (0.96–0.98)	0.95 (0.94–0.95)	1.35 (1.33–1.37)	1.43 (1.42–1.45)
Year	2001	1	1	1	1
	2002	1.10 (1.04–1.16)	1.06 (1.03–1.10)	1.00 (0.81–1.24)	1.01 (0.87–1.18)
	2003	1.27 (1.21–1.33)	1.30 (1.26–1.34)	1.05 (0.87–1.29)	1.08 (0.94–1.25)
	2004	1.32 (1.26–1.38)	1.39 (1.35–1.43)	0.99 (0.81–1.20)	0.98 (0.85–1.12)
	2005	1.34 (1.28–1.40)	1.49 (1.44–1.53)	1.07 (0.88–1.29)	1.04 (0.90–1.19)
	2006	1.35 (1.29–1.42)	1.55 (1.51–1.60)	1.02 (0.85–1.24)	1.02 (0.89–1.17)
	2007	1.45 (1.39–1.52)	1.61 (1.56–1.65)	0.97 (0.80–1.16)	1.04 (0.91–1.18)
	2008	1.45 (1.38–1.52)	1.71 (1.66–1.75)	0.95 (0.79–1.12)	0.96 (0.84–1.09)
	2009	1.45 (1.38–1.51)	1.79 (1.74–1.84)	0.94 (0.78–1.12)	0.91 (0.80–1.04)
	2010	1.50 (1.44–1.57)	1.84 (1.79–1.89)	0.84 (0.70–1.01)	0.94 (0.82–1.07)
	2011	1.61 (1.55–1.68)	1.99 (1.93–2.04)	0.85 (0.71–1.02)	0.88 (0.77–1.00)

CCI: Charlson Comorbility Index IHM: In-Hospital Mortality (OR: Odds Ratio) CI Confidence interval

Therefore, the prevalence of depression in women with diabetes was almost twice as high as 10 years earlier. Among patients with T2DMand concomitant depression, older age and greater comorbidity (Charlson comorbidity index) were significantly associated with a higher risk of dying, in both sexes (Table 5). The time trend analysis (2001–2011) showed no significant change in IHM for men and women with T2DM and depression.

## Discussion

Based on the Spanish National Hospital Database, our results reveal that almost 5% of hospitalized patients with T2DM have an associated diagnosis of depression and that the prevalence of depression increased significantly from 2001 to 2011.

We found an increase in the prevalence of depression over time in both men and women with T2DM; the prevalence of depression was more than 2.7 times higher in diabetic women than in diabetic men. Our multivariate analysis showed that the prevalence of depression increased more in women than in men, a pattern that has also been observed in most population-based epidemiological studies of people with diabetes [2, 22, 23].

Roy et al conducted a systematic review to identify published literature on the epidemiology of diabetes and depression finding that of nine studies that examined differential rates of depression in men and women with diabetes, with the exception of one study, women had higher rates of depression than men [2].

The estimated pooled prevalence obtained by Ali et al. after a meta-analysis was 23.8% among females with diabetes, higher than their male counterparts with diabetes (12.8%) [1].

In Spain a cross-sectional study, conducted in year 2010 among 12,392 T2DM patients found that the prevalence of depression was 5.2% for males and 15.1% for females [5].

Hoffman *et al*. [18] found that depression measured using self-report scales was associated with an increased risk of all-cause mortality (HR 2.56, 95%CI 1.89–3.47). It is logical that the prevalence of depression observed in epidemiological studies using specific diagnostic instruments is 3 to 4 times higher than that calculated based on discharge data. According to the methodology of the Spanish National Hospital Database, only those clinical conditions that affect patient outcome are codified. Therefore, patients whose depression was well-controlled during hospitalization will possibly not have these codes in their discharge report.

Our results suggest that the prevalence of comorbid depression is greatest among younger diabetic adults. Several studies have shown that older age has a protective effect on depression among diabetics [22, 24, 25]. A large population-based study from Taiwan found that the prevalence of diabetes in people with major depressive disorder was especially prominent among people with depression in their thirties, suggesting that lifestyle factors among this age group may be involved [26]. Roy et al reported that a number of community-based studies in the U.S. have shown an increased prevalence of psychological morbidity in younger adults with T2DM [2]. Similar results are have been found in Canada using cross-sectional data from the National Population Health Survey [27]. Furthermore, patients with co-morbid depression and diabetes tend to be younger than patients with diabetes but without depression [15, 28, 29]. Conversely, other authors have reported older age as a risk factor for higher prevalence of depression [30, 31].

Jimenez-Garcia *et al*. (2012) [4] found a negative relationship between age and psychological distress in diabetics in Spain. Younger adults may be more reactive to life stressors, experience chronic disease as more developmentally unexpected, and cope less effectively with these conditions than older adults [32]. Increased physician sensitivity to mental disorders among the younger may also explain this higher prevalence. However other study conducted in Spain has found higher prevalence of depression among older diabetic patients (age >64) than among younger ones [5].

Different methods used in these investigations, including sample selection, setting and diagnosis methods, could explain the heterogeneity of the research literature in this field. Therefore, the relationship between age and risk for depression in people with diabetes remains complicated and needs further exploration. We found a higher prevalence of depression in diabetic women than in diabetic men in all primary admission diagnoses based on disease categories. Current evidence suggests that depression causes a greater increase in the incidence of cardiovascular disease in women, and that the prevalence of depression is higher in women with cardiovascular disease than in men [33].

A recent study conducted by Schoepf *et al*. [34] indicated that T2DM increased prediction of in-hospital death in people with major depressive disorder by 17.1%. In this context, 2 meta-analyses by Park *et al*. [35] and Van Dooren *et al*. [36] report depression to be associated with a 1.5-fold increased risk of mortality in diabetic individuals. However, our results reveal that IHM remained stable over time among diabetic men and women with depression.

Of note, the comorbidity and median LOHS of patients with diabetes decreased significantly during the study period (Tables 2 and 5); therefore, the increase in the prevalence of depression cannot be explained with a worsening medical profile of patients with T2DM. Furthermore, according to the selected primary admission diagnoses based on disease category, we found no significant change over time for IHM among diabetic men with depression, probably because of improved treatment and superior care in these patients [37].

Health care professionals need to be aware of the increased risk of psychological distress and mental disorders, such as depression, in patients with diabetes. We agree with other authors who recommend screening for depression and affective disorders several times per year, perhaps at each clinical visit, particularly in younger adults [4, 38, 39]. Practice guidelines indicate that because patients with diabetes are more likely to be affected by depression, periodic assessment and monitoring of depression and other mental health conditions is an important component of the management of these patients [40].

The strength of our investigation lies in its large sample size and standardized methodology, which has previously been used to investigate diabetes in Spain and elsewhere [41, 42]. Nevertheless, our study is subject to a series of limitations. Our data source was the Spanish National Hospital Database, which contains discharge data on hospitalizations in Spain and uses information the physician has included in the discharge report; therefore, it does not include all the variables in the clinical history. Our results are consistent with those of other studies, which suggest that the difficulties in distinguishing diabetes-related symptoms from depression-related symptoms [3, 43] may be explained by an increase in the number of diagnoses of depression or greater codification of the diagnosis of depression among patients with T2DM. Furthermore, the severity of the disease and the use of medications for depression (insulin and/or oral) are not collected in the database. However all codes included in the database are physician diagnoses, no information from interview data nor questionnaires is collected.

The national database is also limited by its anonymity (no identifying items such as number of the clinical history, the name of the hospital or any ID or code is provided), which makes it impossible to detect whether the same patient was admitted more than once during the same year. In addition, patients who moved from one hospital to another would appear twice.

Nevertheless, this dataset, which was introduced in Spain in 1982, is a mandatory register, and its coverage is estimated to be more than 95% [20].

To date, no validation study to assess unreported diagnosis of diabetes in administrative databases has been conducted in Spain. However, a recent review and meta-analysis by Leong *et al*. (2013) concluded that a commonly used administrative database definition for diabetes had a pooled sensitivity of 82.3% (95%CI, 75.8–87.4) and specificity of 97.9% (95%CI, 96.5–98.8), based on findings from 6 studies with complete data available [44]. While this definition appears to miss approximately one-fifth of cases of diabetes and wrongly classifies 2.1% of non-cases in the population as diabetes, it seems sufficiently sensitive for monitoring prevalence trends in the general population if its accuracy remains reasonably stable over time [44]. Several studies indicated that hospital discharge registers and the use of an administrative database for diagnosis of psychiatric diseases, including depression, were sufficiently sensitive and specific to be used in epidemiological studies [45, 46].

We were unable to calculate diabetes-specific cumulative incidence rates, because no studies in Spain cover blood glucose measurements for the entire population; consequently, no precise estimation of the prevalence of diabetes is available [47].

We did not include data of does under 35 years because the number of persons with T2DM under this age was very small (<20) in all years analyzed and possibly some of those were coding mistakes really corresponding to T2DM [5, 47]. Previous studies conducted in Spain have shown that T2DM is uncommon at younger ages and used this same age group [5, 47]. Concerns have been raised about the accuracy of routinely collected datasets; however, since these datasets are periodically audited, the quality and validity of our dataset has been assessed and shown to be useful for health research [48].

In conclusion, we provide national data on changes in the prevalence of depression among hospitalized patients with T2DM. Our results show that prevalence of depression increased significantly in diabetic patients from 2001 to 2011 even if the health profile and LOHS have improved over this period. Depression is associated with female sex and younger age. Given the rapid increase in the prevalence of diabetes and diagnosis of depression, these findings emphasize the need for further improvement in programs targeted at preventing, monitoring, and controlling depression in patients with diabetes.
