# Correction: Trends in the Prevalence of Depression in Hospitalized Patients with Type 2 Diabetes in Spain: Analysis of Hospital Discharge Data from 2001 to 2011

**DOI:** 10.1371/journal.pone.0162227

**Published:** 2016-08-29

**Authors:** Ana Lopez-de-Andrés, Mª Isabel Jiménez-Trujillo, Valentín Hernández-Barrera, José Mª de Miguel-Yanes, Manuel Méndez-Bailón, Napoleón Perez-Farinos, Carmen de Burgos Lunar, Juan Cárdenas-Valladolid, Miguel Ángel Salinero-Fort, Rodrigo Jiménez-García, Pilar Carrasco-Garrido

There are errors in the Funding section. The correct funding information is as follows: This study was funded by the FIS (Fondo de Investigaciones Sanitarias—Health Research Fund, grant no. PI13/00118, Instituto de Salud Carlos III) co-financed by the European Union through the Fondo Europeo de Desarrollo Regional (FEDER, “Una manera de hacer Europa”). The funders had no role in study design, data collection and analysis, decision to publish, or preparation of the manuscript.
